# Medical Student Training in eHealth: Scoping Review

**DOI:** 10.2196/20027

**Published:** 2020-09-11

**Authors:** Jean-François Echelard, François Méthot, Hue-Anh Nguyen, Marie-Pascale Pomey

**Affiliations:** 1 Faculty of Medicine Université de Montréal Montréal, QC Canada; 2 Research Centre, University of Montreal Hospital Center, Montreal, QC, Canada University of Montreal Hospital Center Montreal, QC Canada; 3 Department of Management, Evaluation and Health Policy School of Public Health Université de Montréal Montreal, QC Canada; 4 Department of Family Medicine and Emergency Medicine Faculty of Medicine Université de Montréal Montreal, QC Canada

**Keywords:** medical education, eHealth, digital health, mHealth, health apps, telehealth, artificial intelligence, electronic health records, programming, internet of things

## Abstract

**Background:**

eHealth is the use of information and communication technologies to enable and improve health and health care services. It is crucial that medical students receive adequate training in eHealth as they will work in clinical environments that are increasingly being enabled by technology. This trend is especially accelerated by the COVID-19 pandemic as it complicates traditional face-to-face medical consultations and highlights the need for innovative approaches in health care.

**Objective:**

This review aims to evaluate the extent and nature of the existing literature on medical student training in eHealth. In detail, it aims to examine what this education consists of, the barriers, enhancing factors, and propositions for improving the medical curriculum. This review focuses primarily on some key technologies such as mobile health (mHealth), the internet of things (IoT), telehealth, and artificial intelligence (AI).

**Methods:**

Searches were performed on 4 databases, and articles were selected based on the eligibility criteria. Studies had to be related to the training of medical students in eHealth. The eligibility criteria were studies published since 2014, from a peer-reviewed journal, and written in either English or French. A grid was used to extract and chart data.

**Results:**

The search resulted in 25 articles. The most studied aspect was mHealth. eHealth as a broad concept, the IoT, AI, and programming were least covered. A total of 52% (13/25) of all studies contained an intervention, mostly regarding mHealth, electronic health records, web-based medical resources, and programming. The findings included various barriers, enhancing factors, and propositions for improving the medical curriculum.

**Conclusions:**

Trends have emerged regarding the suboptimal present state of eHealth training and barriers, enhancing factors, and propositions for optimal training. We recommend that additional studies be conducted on the following themes: barriers, enhancing factors, propositions for optimal training, competencies that medical students should acquire, learning outcomes from eHealth training, and patient care outcomes from this training. Additional studies should be conducted on eHealth and each of its aspects, especially on the IoT, AI, programming, and eHealth as a broad concept. Training in eHealth is critical to medical practice in clinical environments that are increasingly being enabled by technology. The need for innovative approaches in health care during the COVID-19 pandemic further highlights the relevance of this training.

## Introduction

### Background

In 2018, a survey by the Canadian Medical Association showed that approximately “75% of Canadians believe new technologies could solve existing issues in [the] health care system” [1]. In reality, such technologies are continually being developed to address health care needs in diverse fields. For instance, remote medical interventions can enable access to health care in rural areas as well as support diabetes management [2,3]. Medical mobile apps can enhance asthma management [4]. Artificial intelligence (AI) can measure cancer risk or predict mental health outcomes [5,6]. The concept that is defined by the use of such technologies in health care is termed *eHealth* [7]. As the COVID-19 pandemic challenges health care systems worldwide, eHealth technologies enable physicians to continue to provide medical consultations while maintaining social distancing. In this context, clinicians must “conduct more virtual consultations than before, while uncertain about how to do so effectively.” This crisis also highlights the need for innovative approaches in health care [8]. eHealth encompasses many technologies and is not limited to remote medical interventions.

### Defining eHealth

Various definitions of eHealth have been proposed by many authors during the last two decades. The definitions vary in breadth, ranging from being vague to highly specific. According to recent definitions that could be deemed either too broad or too narrow, eHealth is the use of information and communication technologies to enable and improve health and health care services [9]. Various technologies fit into this definition when they are applied to health. This is notably the case for AI, telemedicine, the internet of things (IoT), connected devices, and mobile health (mHealth). Although some may consider the following technologies as part of eHealth, the scope of our definition does not include 3D printing, robotics, blockchain, and nanotechnology. Important terms regarding eHealth are defined in Multimedia Appendix 1.

### Strategic Approaches to eHealth

National eHealth strategies have been adopted by various countries, including Australia in 2008 and France in 2016 [10,11]. The World Health Organization also published a *national eHealth strategy toolkit* in 2012 [12]. According to the aforementioned eHealth strategies, adequate workforce education and training are required and may depend upon “development, integration or changes to existing curricula.” In the same spirit, in 2014, Canada developed a set of eHealth competencies for undergraduate medical education, acknowledging that medical students have to be better prepared “to practice in modern, technology-enabled, clinical environments” [9]. Although such initiatives clearly indicate that there is a need to train the next generation of physicians for future medical practice, it is relevant to examine the education that medical students are actually getting regarding eHealth and how this training is perceived. The literature on this topic is heterogeneous and has not yet been comprehensively reviewed.

### Goal of This Study

This review aims to evaluate the extent and nature of the existing literature on medical student training in eHealth worldwide. More precisely, we approached this study with the following research questions: (1) to what extent and how are medical students being educated about eHealth and (2) what are the barriers, enhancing factors, and propositions regarding this training? This review focuses primarily on some key technologies under the umbrella of eHealth, namely mHealth, the IoT, telehealth, and AI.

## Methods

### Theoretical Framework

We followed the 5-stage framework by Arksey and O’Malley in conducting this scoping review: identifying the research question, identifying relevant studies, screening studies, charting the data, and collating, summarizing, and reporting the results [13]. We also followed guidelines from the PRISMA-ScR (Preferred Reporting Items for Systematic Reviews and Meta-Analyses Extension for Scoping Reviews).

### Identifying the Research Question

As described earlier, this review’s primary focus is to map the literature on medical student training in eHealth, given the continuous development of eHealth technologies in medicine and the importance of adequate education for doctors who will have to work in such an environment. Given the breadth of eHealth, the scope of this review has been narrowed to some key technologies under the umbrella of eHealth; therefore, mHealth, the IoT, telehealth, and AI are the primary focus of this review. However, other technologies directly relevant to the research question are deemed to be of interest for this review.

### Identifying Relevant Studies

A systematic literature search was performed in 4 medical databases (Cochrane Library, MEDLINE [Medical Literature Analysis and Retrieval System Online], Web of Sciences, and the *Journal of Medical Internet Research* [JMIR]: *Medical Education*) using keywords developed through a preliminary search on the review topic. The databases were selected based on their broad spectrum of results, specificity for peer-reviewed articles, and relevance for medical topics. The preliminary search on these databases yielded relevant articles, and these databases were therefore deemed adequate. Similarly, no search for gray literature was done because the scope of this review did not extend to articles that had not been peer reviewed. The keywords were selected to gather results about medical student training in eHealth as a broad concept, and some were specifically added to increase sensitivity for articles regarding AI, the IoT, and mobile apps. Increasing sensitivity for these technologies was considered congruent with the primary focus of this review on a subset of key technologies under the umbrella of eHealth. The search terms used in this review are described in Textbox 1.

The search was first performed in June 2019 and included publications from January 2014 to June 2019. Articles that were more than 5 years old were considered less likely to be informative of the present situation as, in general, eHealth and technology are evolving at a rapid pace; this produced an initial publication count of 1624 studies for review. A second iteration of this search was performed in December 2019 to include articles published since June 2019, and this produced 109 additional studies, resulting in an updated initial publication count of 1733 studies. Only articles published in English and French were included in this review.

**Keywords used for database searches.**

(santé connectée OR m-santé OR santé numérique OR santé digitale OR e-santé OR internet santé OR digital health OR ehealth OR e-health OR drug reference* OR Medscape OR Epocrates OR UpToDate OR medical domotic* OR mhealth app OR mhealth apps OR mhealth OR mhealth device* OR smart health device* OR connected health device* OR smart health apps OR mobile health app OR mobile health apps OR medical app OR medical apps OR smart medical device* OR connected health OR connected medical apps OR connected medical app OR mobile medical app OR mobile medical apps OR connected health app OR connected health apps OR connected medical device* OR m-health OR m-health app OR m-health apps OR m-health device* OR mobile health device* OR mobile health app OR mobile health apps OR smart apps OR smart app OR internet of things OR iot OR ai OR artificial intelligence OR deep learning OR machine learning OR appjam OR app jam OR ia OR intelligence artificielle OR apprentissage profond OR apprentissage machine OR appli* médicale* OR app* médicale* OR lanthier) AND (medstudent* OR med student* OR medical student* OR future doctor* OR future physician* OR curriculum* OR externe* OR externat OR étudiant* en médecine OR medschool OR medical school OR faculté* de médecine OR programme* de médecine OR étude* en médecine OR formation* médicale* OR formation* en médecine)

### Study Selection

Following the removal of duplicates from the initial publication count, inclusion and exclusion criteria (Textbox 2) were applied during the study selection process, which was divided into 2 main phases.

In the first phase, after the removal of duplicates, each of the 1451 remaining articles was reviewed by 1 of the 3 authors (JE, FM, and HN), initially excluding articles if the title and abstract were not related to training in eHealth or to medical students. Full texts were read by JE, FM, or HN when the title and abstract were insufficient to include or exclude a given study. The first author (JE) subsequently screened all studies labeled as *included* using the finalized inclusion and exclusion criteria. This resulted in the selection of 16 studies, including both previously described iterations of the search.

In the second phase, we conducted a backward and forward reference search on the 16 articles selected in the first phase. All 746 newly obtained references were subjected to the same selection process as in the first phase using the same inclusion and exclusion criteria. Nine additional studies were added at the end of this process, including both iterations of the search.

Thus, the final count of studies included in this scoping review was 25. Throughout this whole process, one author (MP) assessed each phase to ensure and verify the accuracy of the work and contributed to the analysis of results.

**Inclusion and exclusion criteria used for study selection.**

Inclusion criteria

- Related to training in eHealth
- Related to the training of medical students. When a study population was not limited to medical students, only data exclusive to medical students were included in this review

Exclusion criteria

- Limited to e-learning of subjects other than eHealth
- Not supported by empirical data, obtained either directly or through a literature review
- Interns, residents, and doctors were not considered medical students as they had already obtained their medical degrees and finished most of their curriculum
- Not published in a peer-reviewed journal
- Published in a language other than English or French
- No access to the full article

### Charting Data (Data Extraction)

We created and used a data extraction grid on a spreadsheet to chart the data from the included studies into different categories including study characteristics, target population, intervention characteristics, data regarding various aspects of eHealth, and other statements regarding the goal of this review (Multimedia Appendix 2). Throughout the charting process, we iteratively revised the extraction grid to refine its components.

### Collating, Summarizing, and Reporting the Results

Results regarding the methodology were thematically subdivided into paragraphs supported by tables as well as figures produced using Microsoft Excel. Findings from included articles were deemed relevant in light of the goals of this review. These relevant findings are presented in the form of tables as we aimed to present an overview of the findings without weighing or aggregating these results. Data regarding methodological characteristics and data regarding relevant findings were not aggregated in a single table because the size of such a table would have hindered readability and interpretability. Critical appraisal of included articles and the assessment of the robustness and generalizability of the findings were not performed for this review.

## Results

### Selection Process

**Figure 1:**
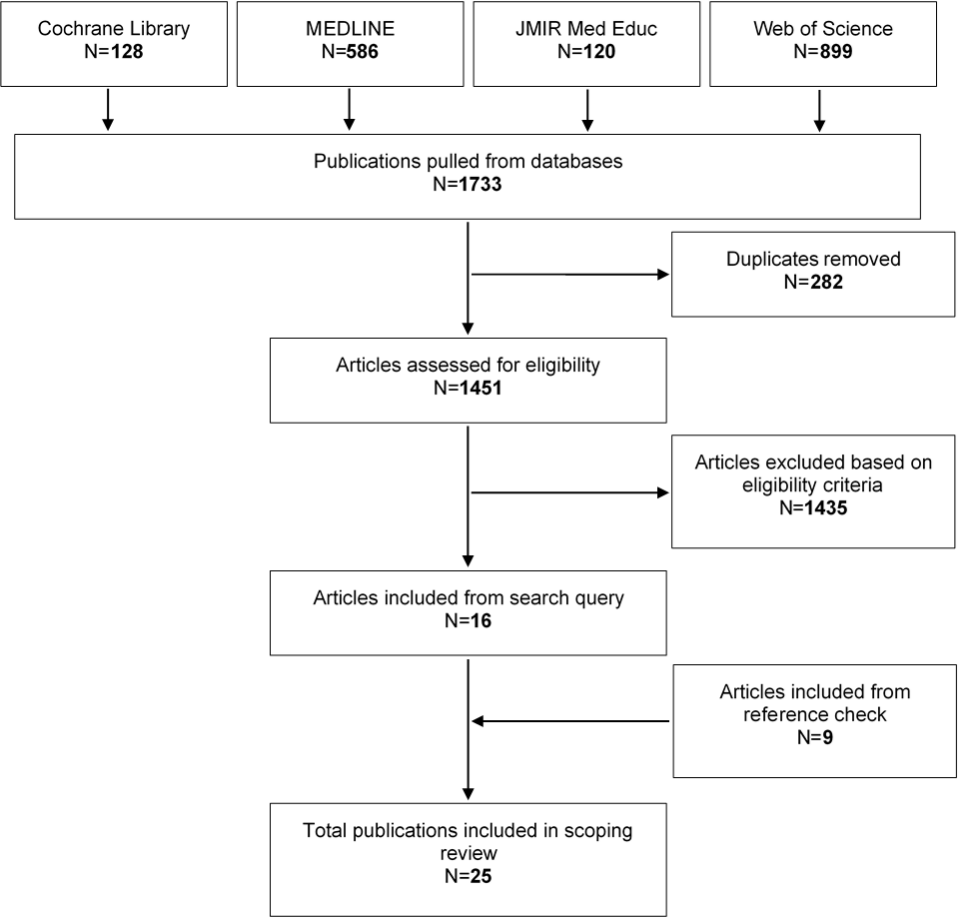
Flowchart of the selection process.

A total of 25 articles were included in this scoping review. The selection process of these articles is detailed in Figure 1, and their characteristics are summarized in Multimedia Appendix 3 [14-38].

### Characteristics of Included Studies

**Figure 2:**
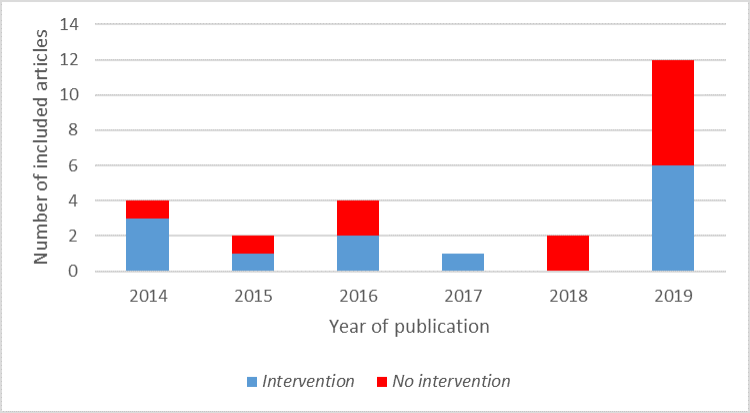
Number of included articles by year of publication and presence of an intervention.

Included studies were published every year from 2014 to 2019. Notably, nearly half (12/25, 48%) were published in 2019. Studies were categorized as *Intervention* (13/25, 52%) or *No Intervention* (12/25, 48%) depending on whether they included an experimental component such as a pilot program. This is summarized in Figure 2.

**Figure 3:**
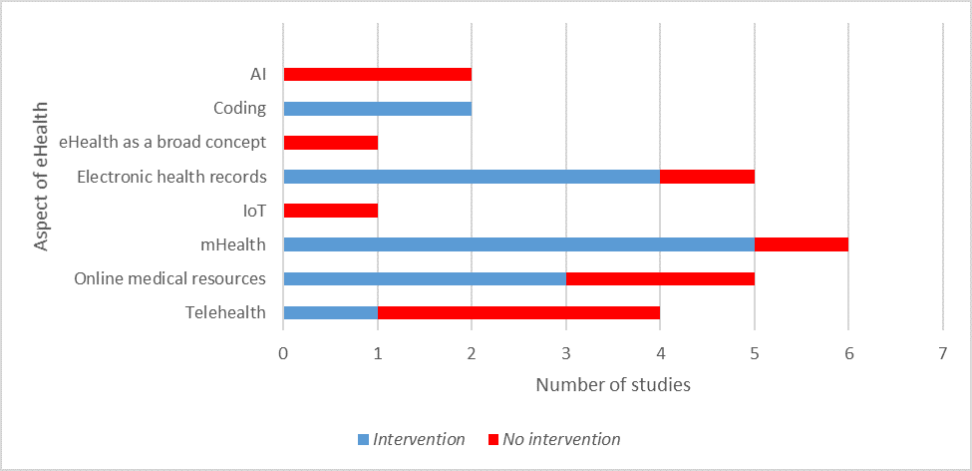
Number of articles discussing each aspect of eHealth. AI: artificial intelligence; IoT: internet of things.

The aspects of eHealth covered by the included studies are summarized in Figure 3. Notably, AI and the IoT were only studied in a *No Intervention* manner, although programming was always studied through interventions.

Of the 20 papers that studied a population of medical students, 11 (55%) had a sample size of more than 100. The smallest study had 9 respondents, and the largest had a population of 17,202. Only half of the studies specified the student’s gender distribution; overall, 55.82% (623/1116) of students whose gender was specified were female. A total of 4 of the included articles had a study population composed of medical school deans, program directors, faculty members, or similarly involved personnel instead of medical students.

Of the studies that did not include an intervention, most consisted of surveys answered by medical students or faculty members. One study was a mixed methods review that complemented a search of the existing literature with interviews conducted with the administration or faculty members of medical schools that included telemedicine in their curricula. Only 1 *No Intervention* study was a pure literature review. Among all the included articles, a few contained quantitative data only (5/25, 20%) or qualitative data only (6/25, 24%) and the majority were conducted with a mixed methodology with both types of data (14/25, 56%). The included studies were published in 17 different journals, with *JMIR Medical Education* (4/25, 16%), *Academic Medicine* (4/25, 16%), the *Journal of Telemedicine and Telecare* (2/25, 8%), and *Medical Teacher* (2/25, 8%) being represented more than once.

Studies were conducted in 12 different countries, with the United States being the most represented. Multiple studies have also been conducted in Canada, Australia, and Germany. The study locations are displayed in Table 1. No included studies were conducted in South America.

Of the 25 included studies, 10 (40%) did not state their sources of funding, if any. Only 4% (1/25) study was funded by a private company, the Western Connecticut Health Network. Another received free UpToDate subscriptions from Wolters Kluwer, but no monetary funding from the company. Moreover, 32% (8/25) explicitly stated that they were not funded, 12% (3/25) were funded by academic institutions, and 12% (3/25) were financed by public American funding agencies.

**Table 1:** Number of studies by study location.

Country	Value, n (%)
United States	10 (40)
Canada	3 (12)
Australia	2 (8)
Germany	2 (8)
France	1 (4)
Oman	1 (4)
Russia	1 (4)
Rwanda	1 (4)
Singapore	1 (4)
Turkey	1 (4)
United Kingdom	1 (4)
Zimbabwe	1 (4)

### Interventions and Main Findings

Of the studies that described an intervention, most measured its effect by assessing the students’ self-reported confidence in using eHealth. Three used more objective methods, either examination results or faculty observation during simulated patient sessions. None of the included articles sought to demonstrate that the eHealth training of medical students objectively influenced care in nonsimulated environments. Most *intervention* studies provided medical students with training and information on eHealth, although only 2 did not. Three studies further provided hardware to medical students, and another provided access to a web-based medical resource. Table 2 contains a summary of all the interventions from the included articles, along with the most relevant findings regarding these interventions. The main findings relevant to this review’s research question for all *No Intervention* articles are presented in Table 3. A summary of the characteristics of all included articles is presented in Multimedia Appendix 3 [14-38], including authors, study location, year of publication, and which aspect of eHealth had been studied.

**Table 2:** Summary of all interventions from the included articles and related findings.

No.	Aspect of eHealth: aim of the study	Intervention and related findings
1	mHealth^a^: to determine whether providing students with preloaded iPad Minis would enhance their experience and increase awareness of and access to mHealth information resources for clinical care in a rural environment	Tablets preloaded with health apps were given to third-year students, who were also asked to complete surveys and a journal An overall positive value for participants who “accessed essential clinical information, experienced improved patient education interactions, and accessed tools and resources to assist them in their experiences” Lessons were learned regarding the project A clerkship director’s request has been made to integrate the project beyond the original pilot
2	Web-based medical resources: to describe the effect of the integration of the OMIM^b^ database during the first year of medical school	The OMIM database was taught to students who later performed self-assessments of short-term and long-term learning Students’ confidence in clinical genetics skills increased after the OMIM education session Acknowledging and incorporating students’ search preferences can engage them in the importance of identifying appropriate resources
3	Programming: to determine whether it is possible to teach medical students the basics of programming in 2 days and whether students value programming and its teaching in medical school	The Coding for Medics course was developed. After 2 days of intensive teaching, participants were given a few weeks to submit a project Basics of programming successfully taught in 2 days Programming teaching should be offered but optional, “practical” and “relevant to clinical problems” Computational thinking learned and considered “transferable” Programming valued as an important skill for the future and oversubscribed because of enthusiasm Programming deemed necessary for the development of eHealth technologies
4	Programming: to describe a new elective computing course and discuss how it prepares medical students to use computer science and technology	A 14-month Computing for Medicine certificate course (C4M) was developed in collaboration with the Department of Computer Science, University of Toronto. The C4M included workshops, seminars, and a project Reinforced valuing and understanding of technology Programming and algorithmic and logical thinking skills were taught Medical schools should consider computer literacy as an essential skill to enhance engagement with technology, collaboration with developers, and patient care quality Questions raised about broader adoption of learn-to-code programs, whether elective or mandatory
5	EHRs^c^: to develop a course module and evaluate it to identify and share best practices and strategies	Mandatory participation in EHR full-day intensive training over 2 days for fifth-year students within their seminar in internal medicine Positive attitude toward EHR usage and software Higher perceived benefits of EHR for doctors and nurses than for other professionals or patients Low perceived benefits of EHR for coworking in multiprofessional team Documentation is a core competency More training, standardized examination, and awareness regarding EHR are needed
6	Online medical resources: to verify the hypothesis that removing the subscription cost barrier to accessing EBCRs^d^ will lead to high student uptake and to an improvement in educational outcomes	Agreement with Wolters Kluwer to facilitate the donations of UpToDate subscriptions to students Access to devices and the internet is not a barrier The focus should be on web-based tools and evidence Higher use of EBCRs when cost barrier removed Lower UpToDate uptake by preclinical students The introduction of EBCRs during the last year of medical school may lead to habit formation Improvement in examination performance of this graduating class Equitable access to information is required
7	mHealth: to allow students to acquire and develop skills using devices and health apps in a clinical context	A single-semester elective option, “Computer Games and Applications for Health and Well-being,” was introduced for first-year students Students not as adept at using mHealth devices as the literature had predicted Ownership of a suitable mobile device was lacking Availability of useful, free apps was limited Key lessons were learned, which we wish will help prepare the medical curriculum
8	Telehealth and mHealth: to deliver orthopedics education through a mobile app, MyDoc, although teaching medical students about secure communication and the Personal Data Protection Act of Singapore	Third-year students were asked to use the MyDoc mobile app that allowed communication in the form of personal messages, case discussions, and sharing of patient details with peers Excellent acceptance and satisfaction Technical issues needed to be addressed There was a need for compliance with privacy laws in the context of the growth of telehealth, so medical schools should consider integrating this secure communication tool to their training
9	Online medical resources: to analyze the effectiveness of a new EBM OSCE^e^ for the end of third-year students	In this OSCE, students were provided with computer stations and performed online searches to answer a standardized patient’s questions An average of 4 search tools were used Most commonly used websites were UpToDate and Google Most students successfully provided the patient with relevant evidence This new OSCE allowed proper assessment of student EBM skill
10	EHRs: to verify the hypothesis that an educational intervention for second-year students improves their ability to use the EHR in a way that enhances patient-provider interaction (EHR ergonomics) during a SP^f^ encounter	EHR ergonomic training’s impact on patient-provider interaction during SP encounters was compared with the impact of basic EHR training with no additional EHR ergonomic training EHR use improved with EHR ergonomic training Students felt improvement in engaging the patients, articulating EHR use benefits, addressing patient concerns, positioning EHR device, and integrating EHR in patient encounter A minimum of 3 ergonomic training sessions were necessary to see overall improvement Self-perceptions were consistent with performance as observed by SPs and faculty members
11	mHealth: to determine whether medical students, with little or no prior knowledge or training in app development, can use development tools to develop useful health apps	Medical students were taught the fundamentals of health app design and development and asked to use the iBuildApp environment to develop an app Perceived need for such training increased Previous programming experience was the strongest influencer of a positive experience It is possible to teach medical students the fundamentals of app design so that they may contribute to health app development
12	mHealth: to determine the ways by which third-year students used mobile technology for learning and clinical decision support	Students were provided an iPad and information was collected with beginning and end-of-year questionnaires, iPad usage logs, weekly rounding observations, and weekly semistructured student interviews over a 12-month period Tablet computers used to enhance patient care and learning in clinical contexts Data service capability and midlevel storage capacity should be provided on each device Quarterly app training should be integrated to increase effectiveness in clinical decision support
13	EHRs: to address a training gap by describing the Sim^g^-EHR curriculum and sharing participant feedback and lessons learned	The Sim-EHR curriculum, consisting of simulated charts for virtual patients, was implanted as part of the third-year family medicine clerkship Increased comfort with finding information, inputting orders, and updating a health maintenance tool Recognition of the value of the activity Expressed frustrations with timing and opportunity costs Improved ability to place orders and update chart No difference in ability to use a health maintenance tool to create routine disease screening, prevention, and management alerts

^a^mHealth: mobile health.

^b^OMIM: Online Mendelian Inheritance in Man.

^c^EHR: electronic health record.

^d^EBCR: evidence-based clinical resources.

^e^EBM OSCE: Evidence-Based Medicine Objective Structured Clinical Examination.

^f^SP: standardized patient.

^g^Sim: simulated.

**Table 3:** Summary of the main findings regarding medical students’ training in eHealth for articles that did not contain interventions.

No.	Aspect of eHealth: aim of the study	Main findings
14	AI^a^: to examine medical students’ perceptions of the impact of AI on radiology, contributing factors, and influence on their choice of specialty	Students believed education on AI is important Students recommended inviting experts Students recommended discussing AI in preclinical radiology lectures AI was not mentioned in the curriculum AI courses and projects were equally effective as formal computer science education More education needed to relieve students’ anxiety and ensure the long-term prosperity of radiology
15	AI: to assess medical students’ feelings on AI in radiology and medicine and to evaluate whether they were worried about AI replacing radiologists and other physicians	Students want AI and deep learning to be incorporated into medical curricula Students need better understanding of deep learning and AI, as well as knowledge of “what data are needed for which type of tasks” and “how AI algorithms should be evaluated” Training will maybe compensate for the tendency of males and more tech-savvy respondents to be more confident, less concerned, and more interested in AI being taught
16	Telehealth: to describe telemedicine education and training implementation and to evaluate the knowledge, attitudes, and practices of deans and associate deans	Telemedicine training implementation was limited compared with mandatory legislation Most respondents expressed a positive attitude toward telemedicine and its potential threats to present medical practices Barriers such as lack of knowledge, resources, support, practice, and funding in telemedicine were identified
17	Telehealth: to analyze the legal, economic, and research-related factors associated with the implementation of telemedicine programs in various countries	Student training in eHealth was one of the factors associated with higher odds of implemented teleradiology and telepathology The average scholarly output related to telemedicine was much higher in countries with versus without training of health care providers
18	IoT^b^: to determine future health professionals' opinions regarding trends in health-related technology, to determine their readiness to use health technologies, and to identify the use of IoT technology in medical applications	Most had no knowledge on the IoT and did not follow publications regarding the IoT Most stated that IoT will affect health, education, genetic and data security, and medical and nursing practices, and that IoT can be used in smart patient follow-ups and mobile health apps Opinions regarding the future of IoT should focus on vital follow-up (blood glucose and electrocardiogram), wearables, and chronic diseases Not aware of the effects of robots or cannot imagine robotic health professionals
19	eHealth as a broad concept: to explore the progress of eHealth training according to curriculum staff and decision makers from all 19 Australian medical schools	All participants knew about eHealth No formal eHealth training programs had been established Informal training and experiential learning during clinical placements were acknowledged eHealth training was considered “important, but not important enough” There were competing curricular priorities, a lack of dialogue with the health system, and no strong drivers for change The situation was unlikely to change until accrediting bodies expect competence in eHealth
20	EHRs^c^: to examine student accounts of EHR use during a time period in which implementation of EHR systems dramatically increased	Students used EHRs in the majority of their clerkships; this use increased from 2012 to 2016 Increase in student entry of information into EHRs Decrease in mean percentage of clerkships in which students entered orders Decrease in student use of paper health records Need to incorporate EHR training into medical school curricula to ensure patient safety and care
21	mHealth^d^: to better understand the experiences in implementing mobile technology initiatives during the clinical years of undergraduate medical education	Eight best practices for introducing mobile technology in the clinical years were identified: plan before implementation, define focused goals, establish a tablet culture, recruit an appropriate implementation team, invest in training, involve students in mentoring, accept variable use, and encourage innovation
22	Web-based medical resources: to examine access, attitude, and training regarding use of electronic resources and EBM^e^ by students after the implementation of the MEPI^f^	Most did not receive formal training in EBM Most who received formal training in EBM found it inadequate Most who did not receive formal training wished to receive EBM training Most did not receive formal training in journal club presentation and scientific reading skills, among which most showed interest in learning these skills Most felt more or less confident in their capabilities of distinguishing the value of medical literature with only 8% (5/61) feeling extremely secure Training required on evaluating sources Inadequate training regarding access to medical literature and information; need to do better
23	Online medical resources: to describe medical students’ behavior and training regarding information search and evidence appraisal	Most reported receiving formal education on information searches This education rarely covered general purpose internet sources, such as Google and Wikipedia, which students use the most EBM summaries were often used and rated higher for accuracy and trustworthiness Bibliographic databases were used the least and rated lower on accessibility and ease of understanding Training on search tools including general purpose internet sources could enhance curriculum
24	EHRs: to determine the amount of time the student spent on EHR use and the potential benefits of student EHR use on education outcomes	Students on the Internal Medicine rotation logged more hours per day on the EHR than students in other clerkships Low EHR use in Obstetrics and Gynecology, Neurology, and General Surgery EHR activity during the Internal Medicine rotation corresponded to half of an average workday No association between self-reported and observed EHR use No correlation between EHR use and patient care based on examinations
25	Telehealth: to characterize medical schools’ approaches for implementing telemedicine training (mixed methods review)	The diverse approaches were a promising sign of accelerating growth in this domain Future effort needed on the part of institutions to make training meaningful and comprehensive Concerns included telemedicine’s inclusion in the curriculum being *cursory* or not *meaningful*

^a^AI: artificial intelligence.

^b^Iot: internet of things.

^c^EHR: electronic health record.

^d^mHealth: mobile health.

^e^EBM: evidence-based medicine.

^f^MEPI: Medical Education and Partnership Initiative.

## Discussion

### Overview of the Literature

This review aimed to evaluate the extent and nature of the existing literature on medical student training in eHealth, while examining what this education consists of, the barriers, enhancing factors, and propositions for improving medical curriculum. This review focuses primarily on key technologies such as mHealth, the IoT, telehealth, and AI. An overview of the literature is discussed in this subsection.

The most studied aspects of eHealth were mHealth, web-based medical resources, electronic health records (EHRs), and telehealth, while eHealth as a broad concept, the IoT, AI, and programming were the least studied aspects. The marked increase in the number of publications on eHealth and medical students in 2019 (6 times more than the previous year) indicates that a greater amount of research has been conducted in the last few years and is likely to signal a larger number of publications in the next few years.

A total of 52% (13/25) of the included articles contained an intervention. Some aspects of eHealth were mostly studied through an intervention; this was the case for mHealth, EHRs, web-based medical resources, and programming. On the contrary, no interventions were part of the methodology of most studies regarding telehealth, AI, the IoT, and eHealth as a broad concept. These results might indicate that some aspects of eHealth are easier to examine or best studied through interventions while others are not. For instance, the IoT and AI were only covered through surveys without any interventions, perhaps because it would be difficult to build a practical yet realistic training program regarding these topics. Programming for medical students was examined through studies containing interventions, which could be attributed to its arguably greater potential for hands-on training (eg, by asking students to develop a simple program) compared with other technologies. Overall, the study population consisted of slightly more females than males, in accordance with the gender distribution of medical students in western countries such as the United States and the United Kingdom [39,40].

### Barriers, Enhancing Factors, and Propositions

The findings of the included studies comprehended barriers, enhancing factors, and propositions for improving medical training in eHealth and may also help researchers formulate other hypotheses on the subject. Identified barriers include competing for curricular priorities, lack of dialogue with the health care system, no strong drivers for change, technical issues (eg, internet access), and limited availability of useful, free items. Enhancing factors include student characteristics (eg, tech-savviness), students’ interest, careful planning, and goal setting. Propositions include implementing new courses and rotations, inviting experts to medical schools, planning better before implementation, mentoring by students, and investing resources.

As there are probably many more barriers, enhancing factors, and propositions that have not been described in the extant literature, we recommend that additional studies be conducted to better identify themes for eHealth as a broad concept as well as for each technology.

### Gaps in the Literature

No study has examined the impact of eHealth training on real health care outcomes, probably because measuring this would be too complicated, long, costly, and subject to many confounding and modulating variables. Only 2 studies directly observed clinical skills related to eHealth, although both times through simulated, standardized patients. These 2 articles evaluated medical student performances with web-based medical resources and EHRs. We therefore recommend that researchers evaluate both learning outcomes and patient care outcomes from training in eHealth.

Less data have also been collected about competencies that future doctors should develop to be ready for medicine that is increasingly digitalized. Therefore, we recommend conducting studies about what knowledge, abilities, and competencies medical students should acquire both in their preclinical and clinical forms.

Stakeholders would especially benefit from a significant increase in the literature on the IoT and AI, although we recommend that additional studies should be conducted regarding eHealth as a broad concept as well as regarding all related technologies.

Our recommendations are detailed in Table 4 in the form of research topics and specific aspects. No specific methodologies for future studies are recommended, but a diversity of study types would probably best enhance the literature. Studies either containing an intervention or not would be relevant.

**Table 4:** Recommendations in the form of research topics and specific aspects.

Research topics	Specific aspects
What is the current state of training in eHealth? (notably regarding the internet of things)	Optimal training Suboptimal training Little to no training Theory versus practical Preclinical versu*s* clinical Optional versus mandatory
What are the barriers to training in eHealth?	Student characteristics (eg, age, prior education) Competing curricular priorities Lack of dialogue with the health care system No strong drivers for change Lack of interest Technical issues (eg, internet access) Limited availability of useful, free items
What are the enhancing factors for training in eHealth?	Student characteristics (eg, age, prior education) Perceived relevance Students’ interest Medical school personnel’s interest Governments and leaders’ interest Medical associations’ interest Strong drivers for change
What could be done to enhance training in eHealth?	Increasing interest of students, medical school personnel, governments, leaders, and medical associations Increasing requirements by accrediting bodies Implementation of new courses Implementation of new rotations Inviting experts to medical schools Planning ahead (eg, anticipating technical issues) Mentoring (by students, residents, and doctors, etc) Investing resources such as funding
What are the competencies and skills in eHealth that medical students want or should acquire?	Knowledge of basic principles Science, technology, engineering, and mathematics Data for surveillance, planning, and managing of scarce resources Data visualization, analysis, quality assessment, and governance eHealth applied to public health and preventive medicine Confidentiality and risks associated with data collection and communication Critical appraisal Technical skills (eg, programming) Cognitive aspects (eg, computational thinking) Interdisciplinary collaboration Communication Ethics and legal aspects
What is the impact of the implementation of an initiative such as a special course or a special rotation related to eHealth?	Learning outcomes Patient care outcomes Students’ appreciation Costs Best if optional versus if mandatory
How is ehealth training discussed in non–peer-reviewed publications?	Broader discussion of this topic Data, perspectives, information that might differ from peer-reviewed articles Most of the specific aspects for other research topics listed above apply

### Limitations

The results of this scoping review are subject to limitations. Articles published before 2014 that might nonetheless have retained relevance were excluded from this review. Conversely, the pressure on medical schools to implement new eHealth training could be so important that even some of the recently included studies might be already obsolete. As definitions of eHealth differ between authors, this review’s scope might also be considered too narrow and therefore exclude relevant technologies, while the unequal number of keywords used for each technology in the initial search might have resulted in increased sensitivity for some aspects of eHealth, such as mHealth. In the same vein, terms such as *health/medical/clinical informatics* could have yielded more relevant articles on eHealth as a broad concept. In addition, including search terms related to EHRs could have yielded more studies on this aspect of eHealth, which would perhaps have allowed for a more detailed overview of medical student training in EHRs and best practices in this area. Overall, the choice of key terms searched has driven the outcome of this review. Furthermore, no hand-searching of printed sources was performed; however, all recent relevant articles on eHealth were assumed to be indexed on the web. Articles from *JMIR Medical Education* could be overrepresented because this journal’s database was used in this review’s study selection process. The selection of databases has probably driven the results of this review overall, but this decision is supported by the relevant results of our preliminary search on these databases as well as their characteristics. Other databases could have been considered including the Education Resources Information Center, which is more focused on education, although it could be considered less specific for medical topics. Similarly, searching the gray literature would have yielded different articles, but the scope of this review is limited to peer-reviewed articles. Finally, the generalizability of our findings might be limited for medical schools in countries not represented among the included articles.

### Conclusions

This review highlights relevant research findings regarding medical student training in eHealth from 25 included articles. Although a definite assessment of the state of medical education in eHealth cannot be inferred from the extant literature, trends have emerged from the included studies regarding the suboptimal current state of eHealth training and the barriers, enhancing factors, and propositions for optimal training of medical students. We recommend additional studies on these themes, but also on what knowledge, abilities, and competencies medical students should acquire at the preclinical and clinical stages of their undergraduate education. Additional studies should be conducted on eHealth and each of the many technologies it comprehends, but more research is especially needed regarding the IoT, AI, programming, and eHealth as a broad concept. We also recommend that researchers evaluate both learning and patient care outcomes from training in eHealth.

The training of medical students in eHealth is critical to their future practice in clinical environments that is increasingly enabled by technology. There is room for improvement in this regard, which will require meaningful changes to their curricula and learning opportunities. How the challenge of medical student training in eHealth will be met will most likely have a significant impact on health care in the near future. The COVID-19 pandemic highlights the relevance of eHealth training as the need for innovative approaches to health care presents itself both as an opportunity and as a challenge.

## Appendix

Multimedia Appendix 1 Glossary.

Multimedia Appendix 2 Components of the data extraction grid.

Multimedia Appendix 3 Summary of characteristics of included studies.

## Abbreviations

AI: artificial intelligence
EHR: electronic health record
IoT: internet of things
JMIR: Journal of Medical Internet Research
mHealth: mobile health
