# Supplementary material for: Medical Student Training in eHealth: Scoping Review
Source: JMIR Med Educ. 2020 Sep 11;6(2):e20027. doi: 10.2196/20027 (PMC7519432; doi:10.2196/20027)
Supplement: Multimedia Appendix 1 [file mededu_v6i2e20027_app1.docx]

**Appendix 1.** Glossary.

| eHealth | The use of information and communication technologies (ICTs) to enable and improve health and health care services. |
| --- | --- |
| Information and communication technologies (ICTs) | Every technological tool or resource used to transmit, store, create, share or exchange information. |
| mHealth | The practice of medicine supported by mobile devices such as smartphones and tablet computers. This includes health apps. |
| Health apps | Software programs that run on mobile devices and serve a purpose related to health or health care services. |
| Artificial intelligence (AI) / Machine learning / Deep learning | The use of computers to perform human-like tasks such as learning, perception and problem solving. Machine learning, a subset of AI, refers to the ability of computers to improve their performance on such a task without being explicitly programmed to do so, by using training data instead. Deep learning is a further extension of machine learning. |
| Telemedicine | The practice of medicine remotely using ICTs, in which the physician and the patient are not in each other’s presence. This includes the use of mobile telephony and the Internet but excludes faxing and postal communication. Telemedicine can be further divided in teleconsultation, teleexpertise, telemonitoring and teleassistance. |
| Internet of things (IoT) | The linking of smart devices and computer systems in order to collect, communicate and exploit data via a wireless network. |
| Connected health devices | Devices collecting physiologic data from the user and communicating this information via a wireless network to a computer system such as the user’s smartphone or their physician’s tablet. |
| Electronic Health Records (EHRs) | Digital, real-time, patient-centered records containing health information available to authorized users. |
