# Supplementary material for: Medical Student Training in eHealth: Scoping Review
Source: JMIR Med Educ. 2020 Sep 11;6(2):e20027. doi: 10.2196/20027 (PMC7519432; doi:10.2196/20027)
Supplement: Multimedia Appendix 2 [file mededu_v6i2e20027_app2.docx]

**Appendix 2.** Components of the data extraction grid.

| **Category** | **Components** |
| --- | --- |
| **Study characteristics** | Title  Author  Journal  Year of publication  Years of data collection  Study location  Type of study (literature review or primary data)  If review   - Search strategy - Inclusion and exclusion criteria - Number of articles included - Characteristics of studies - Data extraction and analysis by authors   Quantitative or qualitative  Retrospective or prospective  Aim of the study |
| **Target population** | Demographics (age, gender, program year)  Size  Response Rate  Technology usage  Informatics literacy  Specialty choice |
| **Intervention studied** | Description, duration and control group |
| **eHealth aspects** | Data related to artificial intelligence applied to health  Data related to mHealth  Data related to connected health devices and the internet of things applied to health  Data related to telehealth  Data related to other aspects of eHealth |
| **Statements** | Regarding the intervention  Regarding medical students’ level of training in e-health  Regarding medical schools’ curricular needs in e-health |
| **Others** | Conflicts of interest & Funding |
