# Supplementary material for: Medical Student Training in eHealth: Scoping Review
Source: JMIR Med Educ. 2020 Sep 11;6(2):e20027. doi: 10.2196/20027 (PMC7519432; doi:10.2196/20027)
Supplement: Multimedia Appendix 3 [file mededu_v6i2e20027_app3.docx]

**Appendix 3.** Summary of characteristics of included studies.

| No. | Title | Authors | Study location | Year of publication | Aspect of e-health | Intervention or No Intervention |
| --- | --- | --- | --- | --- | --- | --- |
| 1 | A library mobile device deployment to enhance the medical student experience in a rural longitudinal integrated clerkship | Johnson and Howard | USA | 2019 | mHealth | Intervention |
| 2 | Bioinformatics for medical students: a 5-year experience using OMIM® in medical student education | Lee-Barber & al. | USA | 2019 | Online medical resources | Intervention |
| 3 | Computer Programming: Should Medical Students Be Learning It? | Morton & al. | United Kingdom | 2019 | Coding | Intervention |
| 4 | Computing for Medicine: Can We Prepare Medical Students for the Future? | Law & al. | Canada | 2019 | Coding | Intervention |
| 5 | Navigating Through Electronic Health Records: Survey Study on Medical Students' Perspectives in General and With Regard to a Specific Training | Hermann-Werner & al | Germany | 2019 | EHRs | Intervention |
| 6 | Better evidence: prospective cohort study assessing the utility of an evidence-based clinical resource at the University of Rwanda | Valtis, Rosenberg, Wachter & al. | Rwanda | 2019 | Online medical resources | Intervention |
| 7 | Lessons learned from piloting mHealth informatics practice curriculum into a medical elective | Fernando & Lindley | Australia | 2017 | mHealth | Intervention |
| 8 | Spaced Education and the Importance of Raising Awareness of the Personal Data Protection Act: A Medical Student Population-Based Study | Daruwalla & al. | Singapore | 2016 | mHealth,  Telehealth (telehealth app) | Intervention |
| 9 | Using standardized patients to evaluate medical students’ evidence-based medicine skills | Amini & al. | USA | 2016 | Online medical resources | Intervention |
| 10 | A Novel Approach to Supporting Relationship-Centered Care Through Electronic Health Record Ergonomic Training in Preclerkship Medical Education | Silverman & al | USA | 2015 | EHRs | Intervention |
| 11 | Health professionals as mobile content creators: Teaching medical students to develop mHealth applications | Masters | Oman | 2014 | mHealth | Intervention |
| 12 | Real-time use of the iPad by third-year medical students for clinical decision support and learning: a mixed methods study | Nuss & al. | USA | 2014 | mHealth | Intervention |
| 13 | Simulated Electronic Health Record (Sim-EHR) Curriculum: Teaching EHR Skills and Use of the EHR for Disease Management and Prevention | Milano & al | USA | 2014 | EHRs | Intervention |
| 14 | Influence of Artificial Intelligence on Canadian Medical Students’ Preference for Radiology Specialty: A National Survey Study | Gong & al. | Canada | 2019 | AI | No Intervention |
| 15 | Medical students' attitude towards artificial intelligence: a multicentre survey | dos Santos & al. | Germany | 2019 | AI | No Intervention |
| 16 | National survey of telemedicine education and training in medical schools in France | Yaghobian & al. | France | 2019 | Telehealth | No Intervention |
| 17 | Worldwide implementation of telemedicine programs in association with research performance and health policy | Avanesova & Shamliyan | Russia (1st author) & USA (2nd author) | 2019 | Telehealth | No Intervention |
| 18 | Perceptions of Turkish health professional students toward the effects of the internet of things (IOT) technology in the future | Bodur & al. | Turkey | 2019 | Internet of things | No Intervention |
| 19 | It’s important, but not important enough: eHealth as a curriculum priority in medical education in Australia | Edirippulige & al. | Australia | 2018 | eHealth as a broad concept | No Intervention |
| 20 | Medical Student Use of Electronic and Paper Health Records During Inpatient Clinical Clerkships: Results of a National Longitudinal Study | Foster & al. | USA | 2018 | EHRs | No Intervention |
| 21 | iPad experience during clinical rotations from seven medical schools in the United States: Lessons learned | Deutsch & al. | USA | 2016 | m-health | No Intervention |
| 22 | Access, attitudes and training in information technologies and evidence-based medicine among medical students at University of Zimbabwe College of Health Sciences | Parve & al. | Zimbabwe | 2016 | Online medical resources | No Intervention |
| 23 | Information-Seeking Behaviors of Medical Students: A Cross-Sectional Web-Based Survey | O'Carroll & al. | Canada | 2015 | Online medical resources | No Intervention |
| 24 | Medical Students and the Electronic Health Record: 'An Epic Use of Time' | Chi & al. | USA | 2014 | EHRs | No Intervention |
| 25 | Telemedicine Training in Undergraduate Medical Education: Mixed-Methods Review | Waseh & Dicker | USA | 2019 | Telehealth | No Intervention (mixed-methods review) |
